# Xanthogranulomatous Pyelonephritis in the Tropics

**DOI:** 10.15190/d.2025.11

**Published:** 2025-09-30

**Authors:** Pallavi Prasad, Sudha Sudha, Gauri Niranjan, Ritu Verma, Jitendra Kumar Vimal

**Affiliations:** Department of Pathology, Sanjay Gandhi Postgraduate Institute of Medical Sciences (SGPGIMS), Lucknow, Uttar Pradesh, India

**Keywords:** Xanthogranulomatous pyelonephritis, nephrectomy, calculi, pathology, pyonephrosis.

## Abstract

Xanthogranulomatous pyelonephritis (XGP) is a rare inflammatory disease caused by chronic urinary tract obstruction or suppuration that occurs in the renal parenchyma. It results in an enlarged, nonfunctioning kidney with diffuse parenchymal damage due to obstructive renal calculi and granulomatous inflammation. Differentiating XGP from renal cell carcinoma, renal tuberculosis, and pyonephrosis can be challenging both clinically and radiologically; therefore, histological investigation after nephrectomy is typically used to confirm the diagnosis. We aimed to study the clinicopathological features of XGP cases confirmed by histopathological examination following surgery. This was a retrospective analysis conducted on 55 nephrectomy specimens diagnosed as XGP over a period of 6.25 years. Clinical details, laboratory and radiological findings were retrieved from the Hospital Information System. The gross and microscopic findings were reviewed. The study included 55 cases, ranging from 6 to 73 years (mean 38 ±18.3 years). The right kidney was involved in 45.5% of patients, and the left was affected in 54.5%. The most common presenting complaint was lumbar/flank pain, followed by recurrent urinary tract infection, fever, hydronephrosis, and an abdominal mass. Surgical interventions included simple nephrectomy (n=48), nephroureterectomy (n=4), radical nephrectomy (n=2), and partial nephrectomy (n=1). Renal/ureteric stones were identified in 49 cases. After histopathological analysis, 40 patients were diagnosed with pure XGPN, 7 with associated chronic pyelonephritis, 3 with hydronephrosis, 2 with pyonephrosis, 1 with nephrocutaneous fistula, 1 with high-grade urothelial carcinoma and 1 with serositis. A positive urine culture was noted in 13 cases. Histologically, all cases revealed sheets of foamy histiocytes, along with a few multinucleated giant cells and chronic inflammation. Cases with associated chronic pyelonephritis were characterised by periglomerular fibrosis, tubular atrophy, thyroidization, and interstitial fibrosis. XGP remains an important mimicker of renal malignancy, both clinically and radiologically. Accurate histopathological diagnosis is crucial to avoid overtreatment and ensure appropriate management. Increased awareness of its clinicopathological spectrum can aid in better diagnostic accuracy and reduce unnecessary radical surgeries.

## 1. INTRODUCTION

Xanthogranulomatous pyelonephritis (XGP) is a rare inflammatory disease caused by chronic urinary tract obstruction or suppuration occurring in the renal parenchyma^1^. The majority of cases are unilateral and result in an enlarged, non-functioning kidney with diffuse parenchymal damage due to obstructive renal calculi and granulomatous inflammation. *Escherichia coli *and *Proteus mirabilis *are considered the primary causative organisms^1^. Studies show that females are more frequently affected than males, with peak prevalence in the sixth decade of life^2^. Common symptoms that patients typically present with include recurrent fever, weight loss or malaise; urinary symptoms such as dysuria or hematuria; and flank pain or a palpable mass^3^. XGP constitutes 0.6–1.0% of cases of chronic pyelonephritis and 19% of those requiring nephrectomy^4^. Even in cases of children who present with perirenal or psoas abscesses, renal masses and/or nonfunctioning kidneys with/without associated urolithiasis, XGP should be considered in the differential diagnosis^2^.

It can be challenging to differentiate XGP from renal cell carcinoma (RCC), renal tuberculosis, and pyonephrosis based on clinical symptoms, physical examination, and imaging; therefore, histological investigation after nephrectomy is typically used to confirm the diagnosis^5^. However, partial nephrectomy or antibiotic therapy can preserve renal function if an appropriate diagnosis can be made before surgery^1^. Histologically, the renal parenchyma is gradually replaced by a diffuse granulomatous inflammatory infiltration that includes xanthomatous cell-macrophages with lipids in their cytoplasm, among purulent secretions. The exact aetiology is yet unknown^6^.

In the present study, we aimed to study the clinicopathological and radiological features of XGP and reviewed 55 cases confirmed by histopathological examination following surgery.

## 2. MATERIALS AND METHODS

This retrospective, single-centre study included radical and simple nephrectomy specimens obtained in the Department of Pathology between January 2018 and March 2024. Follow-up information and clinical and laboratory parameters were obtained by reviewing medical records from the Hospital Information System. The study followed the principles of the Declaration of Helsinki and was exempt from ethical approval The following data were retrieved: age, sex, clinical symptoms (flank/lumbar pain, recurrent urinary tract infection, palpable abdominal mass, nausea or vomiting), side of kidney affected, blood counts, urinalysis, urine culture, use of antibiotics before nephrectomy and type of surgery.

### Pathological analysis

The nephrectomy specimens were fixed in 10% buffered formalin and appropriately grossed as per the standard protocol. Sections were cut at 3‐μm thickness and stained with hematoxylin and eosin stain. Slides from each case were reviewed for detailed morphological analysis, including foamy macrophages, granulomatous reaction, chronic inflammatory cells, giant cells, fibrosis, necrosis, calcification, tubular atrophy, abscess and perinephric fat involvement. Special stains such as periodic acid schiff (PAS) and Ziehl Neelsen were performed as and when required. The imaging details were retrieved from the patient case files.

## 3. RESULTS

Our study included a total of 55 patients (27 males, 28 females). The ages ranged from 6 to 73 years (mean 38 ±18.3 years). Surgical interventions included simple nephrectomy (n=48), nephroureterectomy (n=4), radical nephrectomy (n=2), and partial nephrectomy (n=1). The right kidney was affected in 45.5% (n=25) of patients, and the left kidney was affected in 54.5% (n=30) of patients. The most common presenting complaint was lumbar/flank pain, followed by recurrent urinary tract infection, fever, hydronephrosis, and an abdominal mass (Table 1). Renal/ureteric stones were identified in 49 cases (38 cases had a single stone, 11 had multiple stones). At the time of hospital admission, the various clinical diagnoses were obstructive uropathy (n=49), perirenal abscess (n=2), psoas muscle abscess (n=2), pyonephrosis (n=2), and nonspecific renal neoplasia (n=1). After histopathological analysis, 40 patients were diagnosed with XGPN, 7 with CPN, 3 with HDN, 2 with pyonephrosis, 1 with nephrocutaneous fistula, 1 with muscle-invasive high-grade urothelial carcinoma and 1 with serositis. Thirty-eight patients presented with non-functioning kidney, 4 with poorly functioning kidney, 3 with genitourinary tuberculosis, and 2 with chronic kidney disease.

**Table 1:** Table 1. Demographic characteristics and clinical symptoms

Variables	Value, n (%)
Age in years, mean (SD)	38.4(18.3)
Female : male	3:2
Laterality	
Right kidney	25(45.5%)
Left kidney	30(54.5%)
Nephrolithiasis	
Single stone	38(88.9%)
Multiple stones	11
Absent	6
Clinical presentation	
Flank pain	43(78.2%)
Recurrent urinary tract infections (UTI)	32(58.2%)
Fever	31(56.4%)
Hydronephrosis	2 (3.6%)
Turbiduria	3 (5.5%)
Nocturia	2(3.6%)
Vomiting	3 (5.5%)
Abdominal mass	1(1.8%)

**Figure 1:**
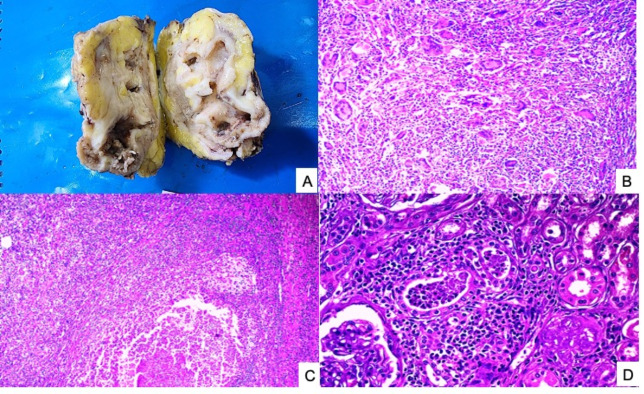
Figure 1. Case of XGPN: (A) Gross of simple nephrectomy specimen showing loss of cortico-medullary differentiation with multiple yellow-white areas. (B) Photomicrograph showing numerous giant cells, (C) focus of neutrophils lying amidst dense chronic inflammation (D).

**Figure 2:**
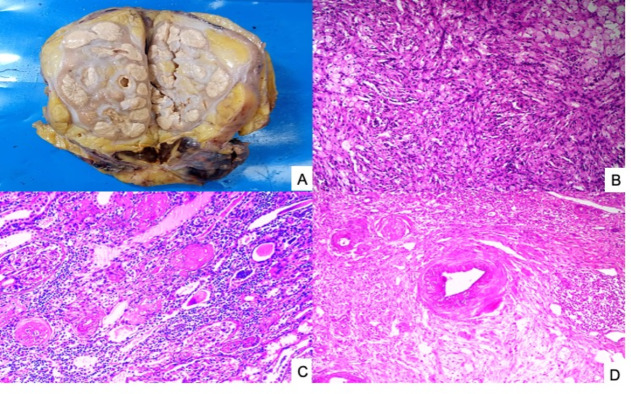
Figure 2. Another case of XGPN misdiagnosed clinically as RCC (A) Gross of simple nephrectomy specimen showing loss of cortico-medullary differentiation with multiple yellow-white nodular areas. (B, C) Photomicrograph showing sheets of foamy macrophages, sclerosed glomeruli and atrophic tubules. (D) Thickened blood vessels were also noted.

Computed tomography (CT) scans revealed segments of low attenuation, hydronephrosis with expansion of renal calyces, parenchymal inflammation and thickening of renal fascia in all patients. Perinephric fat accumulation, a contracted renal pelvis and perinephric/pelvic/post-ureteric stranding were noted in 5, 8, and 5 patients, respectively. Calculi in the renal pelvis were present in 49/55 (98%) patients, 3 of whom had staghorn calculi. Seven patients had ureteric calculi. In two patients, the imaging revealed a renal mass. Gross examination revealed an enlarged kidney with increased perinephric fat in all patients (Figure 1 and Figure 2). The external surface of the adhering renal capsule displayed scarring. Cut-section showed dilated pelvicalyceal system with loss of cortico- medullary differentiation and replacement of renal parenchyma by multiple yellow fatty nodular areas. Seven cases had necrotic regions with grey tan to grey‒yellow patches. In addition, multiple pus-filled abscesses were observed in 2 (3.6%) cases.

On histological examination, the renal parenchyma showed sheets of foamy histiocytes, along with a few multinucleated giant cells and chronic inflammation. Cases with associated chronic pyelonephritis were characterized by periglomerular fibrosis, tubular atrophy and thyroidization, interstitial fibrosis, and chronic inflammation. Renal blood vessel sections were largely unremarkable except for the hyaline arteriosclerosis noted in 18 (32.7%) patients (Figure 1 and Figure 2).

## 4. DISCUSSION

XGP is a rare form of chronic pyelonephritis, with 1.4 cases reported annually per 100,000 people^7^. Although any age group can be affected, middle-aged to elderly patients are most frequently affected. In children, it is diagnosed sporadically and is extremely rare in infants^8^. The mean age varied from 45 to 55.2 years. XGP has a female preponderance^9,10^. Our data shows a female predilection in 50.9% of cases, and the mean age at presentation was 38.4 years. Usually, unilateral lesions affect the right kidney more frequently^6,10^.

In the present study, the left kidney was involved in 30/55 cases, with a predominance of the left kidney being affected in children^8^. Recurrent urinary tract infections and long-term genitourinary obstruction are essential for the development of XGP^11^. 89% of our cases had urolithiasis as the cause of obstruction, the incidence of which was similar to that in one Brazilian study that included 15-year-old patients^6^. Although not a prerequisite, nephrolithiasis, most often with staghorn-type calculi, is a known risk factor for XGP^12^. According to Addison et al., 74.3% of their cases had calculi, of which staghorn calculi were the most common type (51.4%).^13^

The clinical symptoms of XGP are frequently nonspecific, with typical symptoms such as fever, flank discomfort, palpable mass, lethargy, anorexia, weight loss, suggesting nephrolithiasis or chronic pyelonephritis^10,14^. Dysuria, frequency, pyuria, or hematuria may also occur. Abscess formation, fistula formation, and sepsis are known complications. In our case series, 78.2% of cases had flank pain followed by fever (56.4%).

According to the literature, *Escherichia coli *and *Proteus mirabilis *are the two most commonly cultured organisms (35.3% and 17.6%, respectively). *Pseudomonas aeruginosa*, *Klebsiella pneumoniae*, *Serratia marcescens *and *Staphylococcus aureus *are other implicated organisms^10,13,15,16^. 10/13 of our patients were positive for *Escherichia coli *on culture. A previous study reported that the presence of positive urine cultures prior to surgery was a prognostic factor for major complications^17^. Predisposing factors include urinary tract infection, ureteropelvic junction obstruction, severe vesicoureteral reflux, diabetes mellitus, rheumatoid arthritis, cirrhosis, obesity, metabolic syndrome, and depressed immunity^10,18^. Additional findings of pyonephrosis and perinephric abscess were observed in 2 cases and 3 cases, respectively, and one case had a nephrocutaneous fistula. Five patients developed hydronephrosis. Three of our cases had genitourinary tuberculosis. Renal tuberculosis was excluded from the possible diagnoses because of clinico-radiological findings and the natural history of the disease. Laboratory parameters like anaemia, leukocytosis, increased erythrocyte sedimentation rate (ESR), proteinuria, high fasting blood glucose, azotaemia, elevated alkaline phosphatase, increased aspartate aminotransferase, and hypoalbuminemia, can occur but are non-specific^19^. According to a multicenter study by Gauhar et al., laboratory findings included leukocytosis (32.9%), anaemia (72.8%), thrombocytopenia (6.8%) and elevated serum creatinine (24.1%)^20^. Kim et al reported 13/21 cases of leukocytosis (61.9%), and 15/21 cases of anaemia (71.4%)^1^.

**Table 2:** Table 2. Laboratory, radiological, microbiological and CT findings

Variables	Value, n (%)
Urine sediments	
Pyuria (mean +/- SD)	15.1 +/- 25.7
Microscopic hematuria (mean +/- SD)	2.1 +/- 4.2
Hematology	
Hemoglobin g/dl (mean +/- SD)	10.6 +/- 1.8
WBC count per microliter (mean +/- SD)	9.7 +/- 3.9
Positive urine culture	
Escherichia coli	10
Pseudomonas aeruginosa	2
Proteus mirabilis	1
Sterile urine culture	42
CT findings	
Malek 1	37(67.3%)
Malek 2	9 (16.4%)
Malek 3	9(16.4%)

Ultrasound examination in diffuse XGP radiologically reveals fluid-filled masses, hydronephrosis, a restricted pelvis, substantial amorphous central echogenicity, kidney enlargement, and the disappearance of its normal architecture^11^. Ultrasonography findings in focal forms of XGP are mostly nonspecific and have a minor role in patient evaluation. A nonfunctioning or poorly functioning kidney is the most frequent finding on intravenous pyelogram and DTPA renal scans^11^. Computed tomography offers an accurate radiological diagnosis of XGP^10^ and is the cornerstone of diagnostic imaging. The majority of patients (84.6%) who underwent imaging revealed diffuse illness, whereas the remaining 15.4% had localized disease^14^.

Malek and Elder proposed a three-stage radiological categorization for XGP staging based on the extent of the inflammatory process^2^. Three stages of diffuse XGP have been described according to CT findings as per Malek et al: stage 1: limited to the renal parenchyma; stage 2: renal parenchyma and peri-nephric fat; and stage 3: pararenal space/retroperitoneum^15^. In our study, 67.3, 16.4 and 16.4% cases constituted Malek stages 1, 2 and 3 respectively. XGP may be diffuse, focal or segmental^21^.

Preoperatively, XGP presents a diagnostic conundrum because its radiological and clinical symptoms mimic both benign and malignant lesions^22,23^. Histomorphology is pathognomonic with diffuse inflammatory cell infiltration^10^. There is an admixture of lipid-laden foamy macrophages, neutrophils, lymphocytes, plasma cells, and giant cells^9,24^. There is destruction of renal parenchyma by granulomas, abscesses, and lipid-laden macrophages^16^. The histologic differential diagnosis comprises RCC, malakoplakia, megalocytic interstitial nephritis, pyelonephritis, tuberculosis, and perinephric abscess. Benign entities such as malakoplakia and megalocytic interstitial nephritis can be excluded by identifying Schiff-positive, diastase-resistant cytoplasmic periodic acid material in histiocytes. Michaelis–Gutmann bodies are pathognomonic of malakoplakia^10^. Lipid-rich xanthomatous cells may mimic the clear cells of clear cell RCC^16^.

The management of XGP depends on the extent of the disease. Surgery is the current therapy of choice for diffuse XGP. In one study, open nephrectomy was performed in 21 cases (72.41%), laparoscopic nephrectomy in 7 cases (24.13%) and partial nephrectomy in one case (3.44%)^24^. Antibiotics can be attempted as an initial treatment for XGP found in earlier stages with a smaller lesion size^25,26^. Standard treatment comprises a short course of antibiotics followed by nephrectomy^4^. In a three-center study by. Xie et al. reported that >=4 weeks of preoperative antibiotics before laparoscopic nephrectomy for XGPN was associated with a shorter duration of hospital stay and fewer and less severe postoperative complications^27^. Another large multicentre study on 365 patients by Gauhar et al documented urosepsis, recurrent urinary tract infections, increased creatinine, and disease extension to perirenal and pararenal space were independent factors associated with positive bladder urine cultures^20^.

## 5. CONCLUSION

XGP, caused by progressive loss of renal parenchyma and resultant nonfunctioning kidney, is a rare type of chronic pyelonephritis. The unique histomorphology of the specimen aids in the final diagnosis. Selecting the most suitable antibiotic therapy requires knowledge of the most often implicated bacteria and their antibiotic resistance profile, in addition to the clinical presentation and possible severity. Nephrectomy is still the standard treatment for the more prevalent diffuse types.

## Bullet Points

• *XGP is a rare inflammatory kidney disease that often mimics renal cell carcinoma clinically and radiologically.*

• *Histopathological confirmation is essential to prevent misdiagnosis and unnecessary radical surgeries.*

*• Characteristic microscopy includes sheets of foamy histiocytes, mixed inflammatory infiltrates, and necrosis.*
